# Whether medicine supply is really meeting primary health care needs: a mixed-methods study in Shandong Province, China

**DOI:** 10.1186/s41256-024-00374-x

**Published:** 2024-09-05

**Authors:** Zhixin Fan, Tiantian Gao, Qiang Sun, Zaheer-Ud-Din Babar

**Affiliations:** 1 Centre for Health Management and Policy Research, School of Public Health, Cheeloo College of Medicine, Shandong University, Jinan, 250012 Shandong China; 2 NHC Key Lab of Health Economics and Policy Research (Shandong University), Jinan, 250012 Shandong China; 3 Shandong Provincial Hospital affiliated to Shandong First Medical University, Jingwu Road, Jinan, 250021 Shandong China; 4 Centre for Pharmaceutical Policy and Practice Research Department of Pharmacy, University of Huddersfield, Queensgate, Huddersfield, HD1 3DH UK

**Keywords:** Medicine supply, Medicine need, Medicine shortage, Mixed-methods study, China

## Abstract

**Background:**

With the aging population, the increasing prevalence of chronic non-communicable diseases, and the diversified needs for primary health care (PHC) medicines, it is necessary to rethink the functional role of the supply of PHC medicines. This study aims to evaluate the supply of PHC medicines and the status of meeting PHC medicine needs.

**Methods:**

The mixed-methods study was conducted to evaluate the supply of PHC medicines in Shandong Province. In the quantitative study, survey questionnaires were distributed to county hospitals, township hospitals, and patients, and a prescription review was performed in township hospitals. In the qualitative study, semi-structured interviews were conducted with the pharmacy managers, physicians, and patients in county hospitals, township hospitals, and village clinics. A senior pharmacist from a tertiary hospital who has rich experience on the indications for medicine use, accompanied us on a visit to inspect the PHC pharmacies to survey medicine equipment with a professional perspective.

**Results:**

Quantitative analysis revealed that 211 county hospitals and 1,581 township hospitals participated in the survey, revealing the median annual frequency of medicine shortages of 5.0 times for county hospitals and 2.0 times for township hospitals. Of the 6,323 patient medication surveys, after excluding 152 patients not involved in medication use, 945 (15.3%) indicated medicine shortages, with half of these attributable to institutions lacking required medicines (52.8%). On average, the prescription qualified rate of 37 township hospitals was 72.2%. Four final themes emerged during the qualitative data analysis: (1) Supply of PHC medicines; (2) Solutions to the shortage of off-list medicines; (3) Appropriateness of PHC medicines list; (4) Pharmacist workforce development and pharmacy services.

**Conclusions:**

The discrepancy between patients’ need for PHC medicine and present medicine supply is noteworthy. It is suggested that governments should optimize the existing lists to adequately meet patient medicine needs and prioritize medicines for chronic diseases, which is also particularly important for developing countries. Integrated health care may be a novel strategy to establish unified medicines list and achieve uniform pharmaceutical services in PHC.

**Supplementary Information:**

The online version contains supplementary material available at 10.1186/s41256-024-00374-x.

## Introduction

The Sustainable Development Goals (SDGs) by 2030 focus on achieving universal health coverage (UHC), and an important element of UHC is ensuring access to safe, effective, quality and affordable essential medicines [1]. Primary health care (PHC) is widely recognized as the most inclusive, equitable, and cost-effective way of achieving UHC [2]. Integrated health services (IHS), as one of its key components, play an important role in meeting people's health needs throughout their life cycle, including the provision of essential medicines [2].

Countries around the world have made great efforts to ensure access to essential medicines and strive to achieve UHC. For example, Indonesia has introduced the Jaminan Kesehatan Nasional scheme to provide free access to a list of essential medicines for all citizens [3]. Brazil’s Unified Health System comprises the Basic Component of Pharmaceutical Services to ensure adequate availability of medicines, which has stated that cities could create lists of medicines that meet local needs [4]. South Africa has implemented the Central Chronic Medicine Dispensing and Distribution program to improve access to chronic medicines [5].

To improve access to PHC medicines and achieve UHC, China has implemented a series of major reforms. The National Essential Medicines Policy issued in 2009 aims to improve equitable access to essential medicines [6]. The government encourages PHC institutions to stock at least 90% of essential medicines and promotes a “1 + X” system (“1” for essential medicines, “X” for non-essential medicines) to optimize and standardize the structure of medicines [7]. The zero mark-up policy, national volume-based procurement policy (NVBP), and dual invoicing policy have been extended to all public hospitals nationwide to regulate medicine prices and standardize medicine supply [8–11]. These policies have led to a reduction in medicine prices and a further improvement in medicine affordability, benefiting PHC similarly [12, 13].

In addition, under the establishment of hierarchical diagnosis and treatment, China has innovatively applied IHS to optimize and enhance the supply and access to PHC medicines [14]. County Medical Communities (CMCs) represent an effective model for integrating and optimizing health service resources to improve PHC. The CMCs model integrates county hospitals, township hospitals, and village clinics into a coordinated, three-level county medical service system [15]. In 2019, China intensified its efforts to establish CMCs, emphasizing medicine supply as a critical aspect [16]. Within the CMCs, a county hospital serves as the lead unit, encompassing numerous township hospitals and village clinics as members. The lead unit is responsible for the supply and management of medicines within the CMCs. The government encourages the lead unit to formulate a unified medicine list within the CMCs based on the needs of the service area, from which member units procure their medicines. Moreover, the lead unit regularly reviews and consolidates the medicine procurement plans of member units, organizes unified medicine distribution, and oversees unified medicine procurement to enhance the PHC medicine availability. It is also encouraged to organize regular training for pharmacists from member units to promote standardized pharmacy services [17]. These policy initiatives have led to an improvement in the supply of PHC medicines and further promoted the accessibility and affordability of medicines [18–20].

Medicine supply and need are often linked and influence each other. Nowadays, with an aging population and the increasing prevalence of chronic non-communicable diseases (NCDs) [21, 22], China has become the country with the largest elderly population and one of the fastest aging countries in the world. By the end of 2023, there will be nearly 297 million people aged 60 and over, accounting for 21.1% of the total population, with 217 million will be aged 65 and over, accounting for 15.4% of the total population [23]. The high incidence of chronic diseases is another major challenge, with more than 78% of older people suffering from at least one chronic diseases [24]. PHC institutions are gradually becoming the mainstay to treat elderly and chronic patients, leading to a diversification in the need for PHC medicines. Despite gains in China's PHC capabilities, challenges remain, including a lack of PHC workforce, inadequate capacity, and outdated management practices [25]. The ideal role of PHC is to treat chronic, common, and frequently occurring diseases [2]. A large number of high-quality and low-priced medicines have been introduced into the NVBP list [26, 27], providing more choices for PHC medicine supply.

In China, medical institutions are in a stage of self-selection and self-management of medicines. Relevant policies stipulate that medical institutions should establish their own basic medicine supply list and optimize the list based on safe, effective, and economical use of medication, as well as the characteristics of the diseases treated in the institutions [28]. Despite these measures, there is still a need to assess whether the PHC medicine supply is up to date and meets the needs of residents. This requires a comprehensive evaluation involving multiple stakeholders, including medical institutions, physicians, pharmacists, and patients. To ensure a thorough understanding, we conducted a mixed-methods study to evaluate the supply of PHC medicines and the status of meeting PHC medicine needs in Shandong Province, China.

## Methods

### Study design and setting

This mixed-methods study was conducted from July 1, 2023 to January 31, 2024 in Shandong Province, China. Shandong Province, in eastern China, had a total population of 102 million by 2022, making it the second most populous province in China, with 16.7 per cent of the total population aged 65 and above. Additionally, over 50% of older people suffered from at least two chronic diseases [29, 30]. The rural population of this province shares many similarities with other rural areas of China in terms of income levels, literacy, and health.

**Fig. 1:**
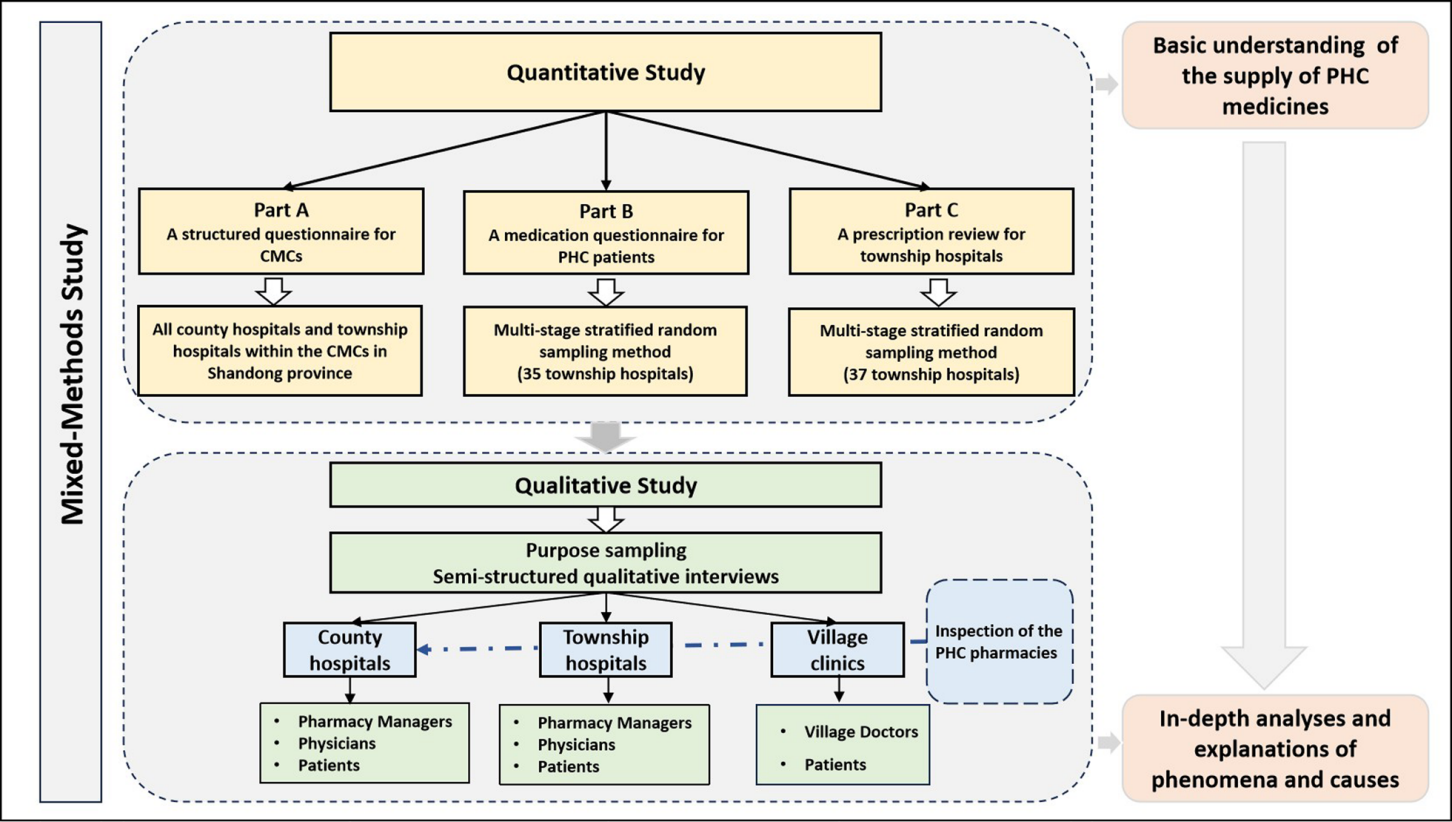
Flow chart of study design

We adopted a sequential explanatory design strategy to first understand the basic situation of the supply of PHC medicines in Shandong Province through quantitative research, and then further in-depth analysis and explanation through qualitative research. The quantitative study consisted of three parts: Part A. a structured questionnaire for all CMCs in Shandong Province; Part B. a medication questionnaire for PHC patients; and Part C. a prescription review for township hospitals. In the qualitative study, semi-structured qualitative interviews were conducted with the pharmacy managers and physicians in county and township hospitals, village doctors in village clinics, and PHC patients. In addition, a professional clinical pharmacist from a tertiary hospital with years of experience and an intermediate title visited county hospitals, township hospitals, and village clinics where participants were located to inspect pharmacies and medicines with a professional perspective during the interview, as shown Fig. 1.

### Data collection and tools

The quantitative study consisted of three parts. In Part A, a structured questionnaire was distributed to all county hospitals and township hospitals within the CMCs with the support and assistance of Shandong Provincial Health Commission. Structured questionnaires included: basic information on medical institutions, medicines supply, pharmacy services, etc. (Appendix 1); in Part B, the study employed a multi-stage stratified random sampling method to randomly select 6 cities in Shandong province based on the level of economic development (high, medium, and low) and geographical location (west, east, and central). For each city, we randomly chose three counties, and then chose three township hospitals within each county. Finally, a total of 35 township hospitals agreed to cooperate with our survey. Two physicians or general practitioners were selected from each township hospital. After the researcher conducted uniform training and pilot testing carried out in a township hospital, a patient survey was carried out on the physician's outpatient shift without any bias in patient selection. Sequentially, medication information from 100 outpatients was recorded, beginning with the first patient. The questionnaire includes: basic characteristics, medication information and medicine shortages for patients (Appendix 2); in Part C, the same multi-stage sampling method mentioned in Part B was used to conduct prescription reviews. Ultimately, 37 township hospitals agreed to participate. In each of the 37 township hospitals, prescriptions were randomly selected from a day in mid-July 2023, with 100 prescriptions in order of number of prescriptions in sequence, without any selection bias. Three clinical pharmacists with clinical professional pharmacist qualification, intermediate title or above, and many years of work experience from tertiary medical institutions in Shandong Province were commissioned to conduct prescription reviews in strict accordance with the Prescription Management Measures.

In qualitative study, purposive sampling was used to select 4 out of 16 cities in Shandong Province, and for each city, a CMC was carefully selected to conduct semi-structured interviews with a county hospital, two township hospitals, and four village clinics under its administrative jurisdiction. The participants were pharmacy managers, physicians, village doctors, and a randomly selected patient who purchased medicine with informed consent during interviews at all levels of the CMCs. All interviews were conducted and recorded in a quiet room in the healthcare facility. Interviews lasted an average of 30 mins (25 to 40 mins). The inclusion criteria for the participants were shown in Table 1. The semi-structured interview guide was developed by clinical pharmacy and public health experts to guide discussions, exploring their understanding of the current state of PHC medicines supply and pharmacy services; whether the supply of PHC medicines truly met patients’ medicines needs and the reason for this (Appendix 3). Semi-structured interviews were conducted by two researchers, one responsible for interviews and the other for audio recording, transcription and time management. To ensure the high quality of the interviews, both researchers were trained in qualitative research and several simulation exercises were conducted before the formal interviews.

**Table 1:** Inclusion criteria for the participants

Participants	Inclusion criteria
Pharmacy managers	1) With a clinical professional pharmacist certificate 2) At least 5 years of practical work experience in the pharmacy 3) Focus on the procurement and storage of medicines and related pharmacy services
Physicians	1) At least 5 years of practice 2) With a clinical doctor certificate 3) As a full-time outpatient physician
Village doctors	1) With a village doctor certificate 2) At least 5 years of practice in village clinics
Patients	1) Over 18 years of age 2) With clarity of expression of views

### Data analysis

In quantitative study, all questionnaire information was double-entered by two researchers using Epidata 3.1 to ensure data completeness and accuracy. Prescription reviews were conducted by two clinical pharmacists simultaneously, and in the event of disagreement, a third party was consulted to ensure consensus was reached, while the results of the reviews were entered using Excel 2017 and verified by another researcher to ensure the accuracy of the data. Count data were described by frequencies and percentages. Tests indicated that the measurement data satisfied the normal distribution, described by means and standard deviations (SD), otherwise, median and interquartile range (IQR).

In qualitative study, recordings were made and converted to text using iFLYTEK software (version SR502, KDDI Ltd., Shenzhen, China). They were then imported into NVivo (version 10) for analysis after assessment by two researchers to ensure accuracy. Two researchers (ZF, TG) analyzed the data for thematic framing based on conceptual content analysis and Colaizzi's method, respectively, to minimize subjective bias and error to ensure consistency in results coding [31, 32]. There were six steps to the data analysis: (1) two researchers seriously examined each interview transcript many times to fully comprehend it; (2) they critically read and scrutinized the transcripts, highlighting sentences and phrases directly associated with the research objectives; (3) the researchers encoded the significant meanings formulated in statements into distinct categories; (4) the categories were grouped into themes, with clusters of themes related to specific questions forming a new theme; (5) each theme was exhaustively described; (6) Post-review, the descriptions were refined for clarity. Any discrepancies in coding were resolved through discussion until consensus was attained. In this study, participant quotations were identified using the coding system. Each quotation was first labelled with the participant number (1–68), followed by the the participant’s role (PM = pharmacy manager, PH = physician, VD = village doctor, PA = patient) and finally, the participant’s workplace (CH = county hospital, TH = township hospital, village clinic = VC). For example, the 1.PM.CH signified a quotation from a pharmacy manager in a county hospital. Data analysis and reporting of qualitative research followed the consolidated criteria for reporting qualitative research (COREQ) checklist [33].

## Results

### Quantitative study

In Part A, questionnaires were returned from 211 county hospitals and 1,581 township hospitals, covering more than 95% of CMCs in Shandong Province. County hospitals were equipped with an average of 774 varieties of medicines, of which 60.0% were essential medicines; township hospitals were equipped with an average of 305 varieties of medicines, of which 76.5% were essential medicines, as shown in Table 2.

**Table 2:** Basic medicines information in county hospitals and township hospitals

Characteristics	County hospital (N = 211)	Township hospital (N = 1581)
Number of medicines varieties (x̅ ± SD)	774 ± 205	305 ± 141
Number of essential medicines varieties (x̅ ± SD)	477 ± 261	236 ± 100
The percentage of essential medicine (x̅ ± SD)	60.0 ± 14.5	76.5 ± 12.1

The median annual frequency of medicine shortages in county hospitals was higher than in township hospitals; the median frequency of medicine procurement in county hospitals was 4.0 days, the same as in township hospitals. And the median of medicine arrival rate in county hospitals was four percentage points higher than in township hospitals, as shown in Table 3.

**Table 3:** Basic information on the medicines supply in county hospitals and township hospitals

Characteristics	County Hospital (N = 211)	Township Hospital (N = 1581)
Annual frequency of medicine shortages (times/year)	5.0 (2.0–10.0)	2.0 (1.0–5.0)
Frequency of medicine procurement (times/month)	4.0 (2.0–4.0)	4.0 (2.0–4.0)
Medicine arrival time (days)	4.0 (3.0–6.0)	3.0 (3.0–5.0)
Medicine arrival rate (%)	92.0 (85.0–96.0)	88.0 (80.0–93.0)

Data is presented in median and IQR

Annual frequency of medicine shortages was based on the medical institution to fill the National Medicine Shortage Reporting System.

Medicine arrival rate = monthly average number of medicines actually arriving/number of medicines on purchase order × 100%;

Medicine arrival time is the monthly average time between the issuance of a purchase order for medicines and the actual delivery of medicines.

According to the results of questionnaire for CMCs, the average percentage of pharmacists with undergraduate degrees or above in county hospitals was 75.3%, compared to 59.3% in township hospitals. Moreover, the educational level of the pharmacists in different medical institutions varied greatly. There were 610 township hospitals (38.6%) with more than 75 per cent of undergraduate degrees and above, while 252 township hospitals (15.8%) had less than 25 per cent. The number of institutions where county hospitals assisted township hospitals in CMC with patient medication counselling and prescription review was 79 (37.5%) and 88 (41.7%), respectively; the number of institutions actually carrying out rotation work at the upper and lower levels was less than 10%.

In Part B, excluding missing information and unqualified questionnaires, a total of 6,323 medication questionnaires were obtained from PHC patients, 51.2% of whom were female and the mean age of patients was 59.6 ± 17.2 years. After excluding 152 patients not involved in medication use, 945 patients (15.3%) reported this medicine shortages, 52.8 per cent of which was due to the facilities not having the required medicines, usually related to the most common diseases such as diabetes, hypertension and coronary heart disease.

In Part C, 125 prescriptions were excluded due to unclear scanning of paper prescriptions or non-medicine prescriptions, we finally conducted prescription reviews on a sample of 3,575 prescriptions. The prescription qualified rate was only about 72.2%, while the average self-reported prescription qualified rate of county hospitals and township hospitals in Part A was 93.8% and 94.2%, respectively.

### Qualitative study

The study interviewed a total of 28 physicians, 12 pharmacy managers and 28 patients. Of the 12 pharmacy managers, 58.3% were women, 75.0% had worked for more than 20 years, and 83.3% had received undergraduate degrees or above. Of the 28 physicians, 17.8% had worked for less than 10 years, and 53.6% had received high or technical school-level education. Of the 28 patients, 64.3% of patients were female, and 71.4% had received junior high school or below education. The mean age of the patients was 53.8 ± 15.2 years, as shown in Table 4.

**Table 4:** Participant characteristics from the interview

Characteristic	Number of pharmacy managers (N = 12)	Number of physicians (N = 28)	Number of patients (N = 28)
Sex			
Female	7 (58.3)	13 (46.4)	18 (64.3)
Age (in years)			
30–39	2 (16.7)	9 (32.1)	4 (14.3)
40–49	9 (75.0)	11 (39.3)	6 (21.4)
50–59	1 (8.3)	8 (28.6)	18 (64.3)
Medical institution			
County Hospital	4 (33.3)	4 (14.3)	4 (14.3)
Township Hospital	8 (66.7)	8 (28.6)	8 (28.6)
Village Clinics	–	16 (57.1)	16 (57.1)
Working experience (in years)			
5–10	1 (8.3)	5 (17.8)	–
11–20	2 (16.7)	8 (28.6)	–
21–30	9 (75.0)	15 (53.6)	–
Highest Education Level			
Junior high school or below	0 (0.0)	0 (0.0)	20 (71.4)
High school or technical school	2 (16.7)	15 (53.6)	4 (14.3)
Undergraduate or above	10 (83.3)	13 (46.4)	4 (14.3)
Practice qualification			
Medical practitioner	–	12 (42.9)	–
Clinical pharmacist	12 (100.0)	–	–
Village doctor certificate	–	16 (57.1)	–

Four final themes emerged during the data analysis: (1) Supply of PHC medicines; (2) Solutions to the shortage of off-list medicines; (3) Appropriateness of PHC medicines list; (4) Pharmacist workforce development and pharmacy services. These themes are described in more detail as follows.

#### Theme 1. Supply of PHC medicines

This theme included two sub-themes that emerged in interviews with pharmacy managers from county and township hospitals and village doctors, including 1) relatively stable supply of PHC medicines based on the existing medicines list; 2) shortage of off-list medicines.

The pharmacy managers in county and township hospitals reported the supply of PHC medicines based on the existing medicines list has been relatively stable and medicine shortages rarely occurred based on the medicine list last year, with shortages of short duration. Even when shortages did occur, they rarely resulted in patients not being able to get their medicines in the existing medicines list when the physicians made prescription, due to the timely checking of pharmacy stocks and advance purchasing plans. Most of the village doctors were very close to the township hospitals and it was very convenient for them to purchase medicines, which were the essential medicines for common and chronic diseases. There were no shortage in the villages.

Medicine shortages are uncommon, but have also occurred a few times in the first few months of this year. Perhaps the medicines of raw materials was out of stock, and then it did not take long to recover, with negligible impact because of the reserve of medicines. (3.PM.TH).

There is no shortage of medicines in our village clinic, and as far as I know, there is no shortage in the surrounding villages either. Most of the medicines we purchase are for chronic diseases, common diseases, and there is no shortage of them. My house is very close to it, and I usually drive as soon as I see we are running out of medicines. (38.VD.VC).

The shortage of off-list medicines based on health outcomes has challenged the supply of PHC medicines. Some pharmacy managers reflected that some patients were referred to PHC by higher-level hospitals for medicine, but PHC did not have such medicines in their medicine list, resulting in a kind of "shortage" of off-list medicines.

In reality, some patients presented us with medicines prescribed by higher-level hospitals that we lack. (6.PM.TH)

#### Theme 2: Solutions to the shortage of off-list medicines

Three themes were included within this theme: (1) recommending alternative medicines; (2) purchasing the required medicines for the patient separately; (3) go to the social pharmacy.

Qualitative results showed when physicians encountered patients who need medicines that were not equipped in the institution, they usually recommended alternative medicines with similar efficacy and price to the patients based on their professional knowledge and clinical experience.

In such instances, we encourage patients to substitute medicines that exhibit comparable clinical efficacy and affordability. Should patients persist, procurement falls to them. (23.PH.CH).

Some healthcare institutions would often purchase the required medicines for the patient separately as a matter of professional ethics.

"For those requiring off-list medicines, we will purchase them individually. They need to wait a few days if they want. (15.PM.TH)

Physicians lacked confidence in their own diagnostic and therapeutic skills, believed that patients were more likely to follow prescriptions from physicians in higher-level hospitals, and were reluctant to recommend alternative medicines to patients, asking them to go to social pharmacies to buy.

#### Theme 3: Appropriateness of PHC medicines list

This theme consisted two sub-themes through qualitative findings analysis: (1) single variety of PHC medicines; (2) medicines list not updated in a timely and dynamic manner. More details were described as follows:

The appropriateness of the medicines list needed to be optimized. Some of the medicines currently available in health facilities were not the best. And the unmet medication needs of patients occurred in PHC. There was a single variety of medicines in their institutions, so that the use of medicines in clinical practice was limited, and sometimes the use of medicines was not based on the actual health needs of the patients. Most of the available PHC medicines were essential medicines to ensure affordability for patients, and some high-quality but slightly more expensive medicines were unlikely to be available in PHC. However, sometimes patients with chronic disease, especially the elderly suffering from multiple chronic diseases, were in need of these medicines. Many of these elderly people were prescribed medicines in tertiary hospitals in their first visit and they were reluctant to change their medicines easily for fear of poor efficacy.

The supply of PHC medicines can basically meet patients' needs for medicines, but sometimes a better medicine should be available for patients, but hospitals are not equipped with it. (31.PH.TH).

Our clinical use of medicines still feels limited. There are some good medicines that are not available in PHC. There are some elderly people with multiple chronic diseases who actually need them. They are very concerned about the efficacy of the medicine if a replacement is recommended (30.PH.TH).

The medicines list was not updated in a timely and dynamically. The physicians after the rotation within the CMCs had revealed certain differences in the medication habits of physicians between the upper and lower levels of hospitals, and there was a certain lag in the equipment and adjustment of medicines in PHC.

Some doctors revealed after the rotation that some medicines had not been used in superior hospitals for a long time, while we still use them, and asked us to change them. (7.PM.CH).

Upon field investigation at healthcare institutions, senior pharmacists reported poor appropriateness of medicines within the medicine supply lists for PHC. Certain medicines, such as compound reserpine tablets and ribavirin injection solution, were removed from professional treatment guidelines or expert consensus. However, these obsolete medicines continued to be favored by PHC.

#### Theme 4. Pharmacist workforce development and pharmacy services

Two sub-themes were included in this theme during the data analysis (as described above): (1) inadequate capacity for pharmacists in PHC; (2) hoping for setting a communication mechanism.

PHC capacity remained inadequate. Pharmacists claimed a necessity for enhanced skills, citing inadequate grasp of recent advancements in clinical medicine, medicine policy changes and inconsistent timely alterations in pharmaceutical application.

Our ability to obtain information is poor, we know little about those clinical guideline updates and national policy changes, and we don't know the medication habits of doctors in higher hospitals. (8.PM.TH).

Pharmacists in PHC hoped for collaboration with superior hospital for guidance and support.

We sincerely hope that there is a mechanism for communication with higher hospitals, either they come down to give us guidance or we go up to ask them for advice and learning, otherwise we can only judge based on our own experience and ability. (10.PM.TH).

## Discussion

The study found that the supply of PHC medicines based on the existing medicines list was relatively stable, and shortages occurred sporadically and locally. However, off-list medicines were in short supply, failing to meet patients' needs as determined by health outcomes, especially for chronic diseases, which are becoming increasingly prominent. In addition, existing medicine supply lists for PHC are poorly adapted, and medicines are not updated and optimized. The development of pharmacist teams and primary service capacity is inadequate and needs to be further strengthened.

In China, the definition of medicine shortage is a clinically necessary and irreplaceable or incompletely replaceable medicine that has been approved for marketing, and is in insufficient or unstable supply for a certain period of time or region [34]. There is a well-established premise that all of these come from the medicine list of medical institutions. They are uniformly submitted to the National Medicine Shortage Reporting System (NMSRS), which is often the most important concern of government policy-makers. Implementing strategies that incorporate dynamic, consistent, and prompt surveillance and early warning, augmented by coordinated reactions, has achieved notable progress, including the development of an integrated platform for acquiring medicine shortage data, a shortage-list management system, and a hierarchical and classified disposal system [35–39].

However, there is another potential shortage: off-list medicines were in short supply, failing to meet patients' needs as determined by health outcomes, which is beginning to challenge our existing supply of PHC medicines. In China, this potential shortage is reflected in PHC in the form of a mismatch between upper and lower levels of medication, where patients are diagnosed at a higher level of care and return to a lower level for their medicines, only to find the required medications unavailable. Several studies have shown that this phenomenon occurs in other parts of China and is a growing concern for government policy-makers [40–42]. It is worth noting that even if there is an adequate supply of medicines on the medicines list of healthcare institutions, which means there is no shortage of medicines, when healthcare institutions do not equip the medicines needed by patients, there is still the embarrassing dilemma of “shortage from the patient's perspective”. Such potential shortages are often invisible and difficult to detect. This is because when a patient has an unmet need for medication, they often choose to replace the medicine or return to the higher hospital to receive it in the clinic. For the former, this strategy is often appropriate for emergency patients, but for patients who require long-term medication, the uncertainty of the efficacy of substitute medicines and medication qualification requires us to seriously consider this behavior; for the latter, PHCs are unable to retain patients because of unavailable medicines for patients, and the resulting outflow of patients runs counter to the national reform objective of promoting hierarchical diagnosis and treatment, which is not what we want to see.

Additionally, another overlooked yet profound issue calls for reconsideration—is the current list of PHC medicines optimally fitted to present needs? Our study found that the appropriateness of medicines on the PHC medicines list is poor. Feedback on the need for common medicines suggests that some have been removed from the list by treatment guidelines or expert consensus in the relevant specialty, but some primary care providers are still accustomed to using them. We hypothesized that potential health effects exist for patients, as no studies have been conducted and adequate empirical evidence is lacking. These issues force us to rethink the functional role in the supply of PHC medicines, which can be transformed from "affordable and accessible" to "better supplied and used" for common and frequent diseases and chronic diseases. This is also in line with the development of hierarchical diagnosis and treatment in China, which promotes the sinking of patients into PHC.

There are two key issues here: first, the development and adaptation of the national essential medicines list (EML); and second, the development and adaptation of the medicines supply list for medical institutions. For the former, the national EML is the cornerstone and an important reference for medical institutions at all levels to select their own medicines supply list, which is directly related to the accessibility and affordability of medicines for patients; for the latter, medical institutions have become the 'last mile' in terms of being able to deliver appropriate medicines to patients. In the past, we have tended to focus on the safety, efficacy, accessibility and affordability of medicines in the existing medicines list, while ignoring the gap between the medicines list and the real needs of patients. PHC medicine supply lists are typically based on the national EML, and we need to rethink whether EML can truly meet the medicine needs of patients. This dilemma is not unique to China, as studies in Brazil, Sri Lanka, Vietnam have shown that EML did not meet patients' needs, especially for chronic diseases [43–46].

Currently, the epidemiology of developing countries is in transition from infectious and parasitic diseases to chronic diseases. However, health systems have not yet adapted sufficiently to cope with the long-term nature of chronic diseases. A study of the availability of medicines in 30 developing countries found that medicines for chronic diseases were less available than for acute diseases, especially in the public sector [47]. With an aging population and an increasing prevalence of chronic disease, the elderly chronic disease population often has multiple commodities or complications, resulting in a more diverse range of medication needs. Inadequate availability of medicines in PHC may hinder the treatment and control of disease [48]. This is an enlightenment for the World Health Organization and even countries around the world, especially developing countries, to develop their EML. It is suggested that governments should optimize the supply of medicines through their public health systems, especially prioritizing for chronic conditions, to ensure that people have access to the treatment they need. The medicines used to treat common chronic conditions should be available in sufficient quantities in any health system.

Nowadays, the development and adaptation of medicines supply list for medical institutions is at the stage of self-selection and self-management. It may be a challenge for PHC to adjust their medicines supply list to meet the needs of patients in their service area and to adapt to the local disease spectrum. A survey of the availability of essential medicines at PHCs in Indonesia indicated that the most common reason for the unavailability of medicines was that they were subjectively deemed unnecessary by PHC staffs [49]. In the process of establishing the CMCs, it is necessary to truly develop a medicines list that meet the medication needs of patients within the CMCs, and to achieve the unification of the medicines list among counties, townships and villages is what we need to work on. China has begun to recognize the problem of the linkage between the upper and lower levels of medication and has proposed to explore the establishment of a unified medicines procurement list within the CMCs [50, 51]. It is important to note that the unified medicines list within the CMCs is not simply adding and deleting medicines list of institutions at all levels, but rather an innovation to promote higher-level institutions to drive lower-level institutions to optimize and improve the level and habits of medication, so as to achieve homogeneous articulation of medication within the county.

It is crucial to recognize that front-line health care providers, including pharmacists, are the ultimate executors of our policies. A persistent weakness in PHC is poor capacity for health service providers. Although this issue has been improved dramatically, it still requires further development [25]. This highlights the importance of pharmacy services. A study from Brazil suggests that the low level of implementation of pharmacy services may be closely related to the low availability of PHC medicines [52]. Under the construction of CMCs, a mechanism of assistance and exchange between medical institutions at the upper and lower levels should be really established. Through substantive exchanges such as pharmacy checkups, medication guidance, and rotation of upper and lower levels, the upper level can lead to an improvement in the capacity of the lower level, and UHC will be achieved early.

This study has several strengths. First, we collected comprehensive information on the supply of PHC medicine from medical institutions in Shandong Province, which are usually not easily accessible, to accurately grasp the current supply of PHC medicine in Shandong Province. Second, we conducted this survey from the perspective of pharmacy managers, physicians and patients at primary healthcare institutions at all levels to multi-dimensional evaluate the supply of PHC medicines and the status of meeting medicine needs. Third, a professional clinical pharmacist visited county hospitals, township hospitals and village clinics where participants were located to inspect pharmacies and medicines from a professional point of view, which is better than most other studies.

Our study also has several limitations. The generalizability of our study is limited by only focusing on Shandong province in China. Our comprehensive literature review has revealed that equivalent scenarios were observed in various Chinese regions. We believe that the outcomes of this research generally illustrate common issues within supply of PHC medicine in China. Second, this study focuses on drawing the attention of government policy-makers and researchers to the gap between essential medicines list and patients' actual medicines needs, but does not provide an in-depth analysis of this gap, which needs to be studied and researched in the future. We believe that this is an important reference for governments, especially in low- and middle-income countries, to formulate essential medicines list.

## Conclusions

The supply of PHC medicines based on the existing medicines list has been relatively stable. However, the shortage of off-list medicines based on health outcomes has challenged the supply of PHC medicines. The fundamental reason for this is the mismatch between the actual patient needs for PHC medicine and the existing medicines supply. We need to rethink the focus on the supply of PHC medicines from existing list-driven to prioritizing patient health outcomes to adequately meet patient medicines needs, especially for chronic diseases. Integrated health care provides us with a new way of thinking, by unifying medicine list in the CMCs, breaking down the barriers between medicine list of different levels of medical institutions to meet the needs of patients in the region, and strengthening the capacity of PHC, so as to effectively promote people's health and achieve the UHC early.

## Supplementary Information

Additional file 1: Appendix 1. Structured questionnaire for medical institutions in the CMCs.

Additional file 2: Appendix 2. Medication Questionnaire for Patients in PHC.

Additional file 3: Appendix 3. Outline of interview with Pharmacy Manager, Physicians and patients.

## Data Availability

The datasets used and/or analyzed during the current study are available from the corresponding author on reasonable request.

## Abbreviations

SDGs: Sustainable development goals
UHC: Universal health coverage
PHC: Primary health care
NVBP: National volume-based procurement
IHS: Integrated health services
CMCs: County Medical Communities
NCDs: Non-communicable diseases
COREQ: Consolidated criteria for reporting qualitative research
IQR: Interquartile range
SD: Standard deviations
NMSRS: National Medicine Shortage Reporting System
EML: Essential medicines list
