# Supplementary material for: Whether medicine supply is really meeting primary health care needs: a mixed-methods study in Shandong Province, China
Source: Glob Health Res Policy. 2024 Sep 5;9:32. doi: 10.1186/s41256-024-00374-x (PMC11375931; doi:10.1186/s41256-024-00374-x)
Supplement: Supplementary file 1 — Additional file 1: Appendix 1. Structured questionnaire for medical institutions in the CMCs. [file 41256_2024_374_MOESM1_ESM.pdf]

Structured questionnaire for medical institutions in the CMC

Date:_______________ Medical Institution:____________

Part 1. Basic information

1. Type of hospital: County Hospital ☐ Township Hospital ☐
2. Level and grade of the institution： _______________
3. Population served by the institution： _______________
4. Average annual number of outpatient visits： _______________
5. Average annual number of outpatient visits： _______________

Part 2. Medicines supply

1. As of 30 June 2023, the number of medicines available in the institution:

   Medicine varieties： ________ Medicine products:________

Note: Calculate the number of varieties according to the generic names of drugs with dosage forms, e.g. Nimodipine Tablets, Nimodipine Capsules, Nimodipine Extended-Release Tablets, Nimodipine Injections are counted as 4 different varieties respectively, but Nimodipine Tablets with different specifications are considered to be 1 variety; Calculate the number of products according to the different manufacturers, and different specifications are counted individually, i.e. each drug with an independent YPID code is counted separately as one Product.

2. The number of essential medicines institution equipped:

   Medicine varieties： ________ Percentage share:________

Note: Basic medicines with the dosage form of the generic name of the drug varieties statistics. Pay attention to the basic medicines to the specifications, as long as the specifications have been equipped with the varieties of medicines, regardless of whether there are no other specifications, the number of varieties of the number of varieties of basic medicines are calculated as a varieties.

3. Average frequency of medicines shortages (times/year)： ________
4. Frequency of medicines procurement (times/month)： ________
5. Average medicines arrival time (days)： ________
6. Average medicines arrival rate (%)： ________

Note: Average medicines arrival rate = monthly average number of medicines actually arriving/number of medicines on purchase order × 100%;
Average medicines arrival time is the monthly average time between the issuance of a purchase order for medicines and the actual delivery of medicines

Part 3. Pharmacy services

1. The total number of pharmacy professionals and technicians: ________, including

Title:

Junior ________ Intermediate _______ Senior ________

Highest Education Level:

College and below _____ Undergraduate _____ Postgraduate and above _______

2. Does the institution carry out prescription review: Yes ☐ No ☐

If answer is yes:

The average number of prescription reviews conducted per year_____time/year

The total number of prescriptions reviewed:_______.

The average prescription qualified rate :_______.

3. Does the institution provide patient medication counselling: Yes ☐ No ☐

If answer is yes:

The average number of people who receive patient medication counselling per year:_______.

4. Does the institution send pharmacist to participate in pharmacy-related training: Yes ☐ No ☐

If answer is yes:

The time of pharmacy training sessions per year:_______.

The number of participants per year:_______.

5. Are there any pharmacy personnel in the CMC who rotate to this institution: Yes ☐ No ☐

If answer is yes:

What is the specific practice of rotation?_____________________________________
