# Supplementary material for: Whether medicine supply is really meeting primary health care needs: a mixed-methods study in Shandong Province, China
Source: Glob Health Res Policy. 2024 Sep 5;9:32. doi: 10.1186/s41256-024-00374-x (PMC11375931; doi:10.1186/s41256-024-00374-x)
Supplement: Supplementary file 2 — Additional file 2: Appendix 2. Medication Questionnaire for Patients in PHC. [file 41256_2024_374_MOESM2_ESM.pdf]

Medication Questionnaire for Patients in PHC

Date:______________　　　　　　　　　**Physician:______________**

Part 1.　Introduction and explain the purpose of the study.

Thank you for agreeing to take part in the survey. We are conducting a research on the project "Primary Level Medicine Supply Security in Shandong Province", which aims to understand the current situation of primary level medicine supply security in Shandong Province and to make policy recommendations to further improve primary level medicine supply security. Your support and cooperation is crucial for the successful completion of the research, and we hope you will fill in the form accurately according to the real situation. Participation in the survey and research is completely voluntary, and you can ask us any questions or comments you may have at any time. We promise that the content of the answers (especially information about personal circumstances and opinions) will only be used for research purposes and will be kept strictly confidential, so you can rest assured. Do you agree to participate? If you agree, please sign to confirm. Thank you for your cooperation!

- Do you agree to participate?　Yes☐　No☐　Sign:________________

Part 2. Demographic Information

1. Number:________________
2. Sex: Male☐　Female☐
3. Age:________________
4. Location:_______city_________country____________village
5. Patient is:

First visit ☐ Repeat visit ☐ Transferred to our hospital from other medical institutions☐

6. Whether the patient needs medication at this visit:

Yes☐ No - excluded from this study☐

Part 3. Patient Medication Information

1. The therapeutic medications needed for this visit are:

Name of medicine:_______ Dosage form:_______ Specification:_______

Whether to limit the production enterprise:_______ Name of Manufacturer:_______

Whether equipped with: Yes☐ No☐

2. Patient's reason for choosing the above therapeutic medicines (single choice, if multiple medicines are used at the same time, judged by the most important therapeutic medicine at this visit):

☐ New treatment plan formulated by the doctor at this consultation according to the patient's condition

☐ The patient was referred for the first time for medication based on the treatment plan formulated after diagnosis at a higher level hospital.

☐ Patient's long-term follow-up in this unit to take medication

☐ Other, please specify: ____________________

3. The treatment duration of the patient using the above medicines (single choice, if multiple medicines are used at the same time, calculate according to the medicine used for the longest period of time):

Within 1 week ☐ 1 to 4 weeks☐ 1 to 3 months ☐

More than 3 months ☐ Lifelong medicine taking☐

3. Whether the above medicines are available in the institution:

Yes ☐ No - Continue to fill in the next part☐.

Part 4. Survey of medicine shortages

1. The reason for the occurrence of the above medicines not being equipped is (single choice):

☐This institution is not equipped with the medicine species

☐The institution was already equipped with the medicine, but it was out of stock recently.

☐The institution is equipped with medicines of the same generic name produced by different companies.

☐The institution is equipped with medicines with different generic names that can be substituted for each other.

☐Other, please specify: ____________________

2. For medicines that are not equipped, the doctor's recommendation is (single choice):

☐Advise the patient to replace the medicine with the same generic name produced by a different company

☐The patient is advised to replace the medicine with a medicine with a different generic name but with the same therapeutic effect.

☐Advise the patient to buy the medicine elsewhere

☐Other, please specify:____________________

3. The patient's choice for the medication that is not equipped is (single choice)

☐Accept the doctor's recommendation to purchase the replacement medication

☐Intend to go to a higher level of care for the medication

☐Intended to go to a social pharmacy to purchase the medication

☐Patient left without purchasing medication, exact decision unknown
