# Supplementary material for: Whether medicine supply is really meeting primary health care needs: a mixed-methods study in Shandong Province, China
Source: Glob Health Res Policy. 2024 Sep 5;9:32. doi: 10.1186/s41256-024-00374-x (PMC11375931; doi:10.1186/s41256-024-00374-x)
Supplement: Supplementary file 3 — Additional file 3: Appendix 3. Outline of interview with Pharmacy Manager, Physicians and patients. [file 41256_2024_374_MOESM3_ESM.pdf]

Outline of interview with patients

Thank you for agreeing to take part in the survey. We are conducting a research on the project "Primary Level Medicine Supply Security in Shandong Province", which aims to understand the current situation of primary level medicine supply security in Shandong Province and to make policy recommendations to further improve primary level medicine supply security. Your support and cooperation is crucial for the successful completion of the research, and we hope you will fill in the form accurately according to the real situation. Participation in the survey and research is completely voluntary, and you can ask us any questions or comments you may have at any time. We promise that the content of the answers (especially information about personal circumstances and opinions) will only be used for research purposes and will be kept strictly confidential, so you can rest assured. Thank you for your cooperation!

Part 1. Demographic Information

1. Number:________________
2. Sex: Male☐ Female☐
3. Age:________________
4. Location:_______city_________country____________village
5. Highest Education Level:

Junior high school or below☐ High school or technical school☐ Undergraduate or above☐

Part 2. Research Questions

1. Do you suffer from chronic diseases? If you take medication for a long time, how often do you buy medication? Where do you usually choose to buy medicine? Do you find it convenient to purchase medicine? (distance, time)

2. Do you think the financial burden of buying medicine is heavy? What is the approximate reimbursement rate of medical insurance? Can the current supply of medicines at the PHC meet your needs for medicines?

3. Have you ever encountered a situation where you could not buy medicines? What is the name of the medicine? How did you solve the problem? (What is your attitude if the doctor recommends a substitute medicine?) How often does this happen?

4. Do you think there is any change in the way you purchase medicines nowadays compared to the past? If so, in what way?

5. What do you think are the problems faced by the people in purchasing medicines nowadays? What are your suggestions and ideas.

Outline of interview with Pharmacy Manager

Part 1. Demographic Information

1. Number:_______________

2. Sex: Male☐ Female☐

3. Age:_______________

4. Location:_______city_________country____________village

5. Highest Education Level:

Junior high school or below☐ High school or technical school☐ Undergraduate or above☐

6. Medical institution where you work:

County Hospital☐ Township Hospital☐ Village Clinics☐

7. Working experience (in years):

5-10years☐ 11-20 years ☐ 21-30years ☐

8. Do you have clinical pharmacist qualification? Yes☐ No☐

Part 2. Research Questions

1. Can you talk about the situation of medical service in primary care institutions?

2. Can you describe your specific job responsibilities?

3. Do you think the institution is adequately equipped with medicines? In your daily treatment, do you often run out of stock of medicines when formulating treatment plans for patients? How often? What is the degree of shortage of basic medicines and medicines under medical insurance? What are your measures to deal with the shortage of medicines? What do you think are the reasons for the shortage of medicines? What do you think can be done to solve the above problems?

4. Approximately how long is the delivery cycle of the distribution company? How long is the institution's repayment cycle? If it is long, what are the reasons?

5. How do you consider the types and quantities of medicines you purchase in your work? What is the proportion of essential medicines? Do you think the current ratio of basic medicines meets the needs of grassroots patients?

6. What do you mean by shortage of medicines? How do you think the shortage of medicines in this village health centre compares with that of its counterparts? What are the reasons for the regional differences? Do you think there is a change in the shortage of medicines compared to previous years? What might be the reason for this?

7. In your opinion, does the current guarantee of medicine supply at the grassroots level meet the demand for medicines at the grassroots level? Is there any difference in the degree of satisfaction for patients with different diseases? What do you think are the reasons?

8. Do you think the current medicine prices are reasonable? Are there obvious

differences in the price fluctuations of different categories of medicines?

9. Are pharmacy services available in your organization? What forms of pharmacy services? Do you think these services have an impact on patient medication? Can you give specific examples. Do you think there is a need to improve the pharmacy services in your organization? Why? Do you think the number of pharmacy technicians in the organization is reasonable (quantity, structure)?

Outline of interview with Physicians

Part 1. Demographic Information

1. Number:_______________

2. Sex: Male☐ Female☐

3. Age:_______________

4. Location:_______city_________country____________village

5. Highest Education Level:

Junior high school or below☐ High school or technical school☐ Undergraduate or above☐

6. Medical institution where you work:

County Hospital☐ Township Hospital☐ Village Clinics☐

7. Working experience (in years):

5-10years☐ 11-20 years ☐ 21-30years ☐

8. Do you have clinical pharmacist qualification? Yes☐ No☐

Part 2. Research Questions

1. From the perspective of your department, do you think that the current supply of medicines meets the needs of the population? Are there differences in meeting the needs of patients with different diseases? What do you think are the reasons for this?

2. Does your organization provide pharmaceutical services? What types of pharmacy services do you provide? Do you think these services have an impact on patients' medication use? Can you give specific examples? Do you think there is a need to improve pharmacy services in your organization? If so, why?

3. Do you think there are enough medicines in the institution? In your daily work, do you often run out of medicines when formulating treatment plans for patients? How often? What is the extent of shortages of basic medicines and medicines covered by health insurance? What measures do you take to deal with medicine shortages? What do you think are the reasons for medicine shortages? What do you think can be done to solve the above problems? From a management improvement perspective, what aspects of the organization do you think would help reduce the incidence of medicine shortages?

4. What do you mean by medicine shortages? How do you think medicine shortages in your organization compare with those of your peers? What are the reasons for regional variations? Do you think medicine shortages have changed compared to previous years? What are the possible causes?
